# A Novel Hepe-Like Virus from Farmed Giant Freshwater Prawn *Macrobrachium rosenbergii*

**DOI:** 10.3390/v12030323

**Published:** 2020-03-17

**Authors:** Xuan Dong, Tao Hu, Qingyuan Liu, Chen Li, Yani Sun, Yiting Wang, Weifeng Shi, Qin Zhao, Jie Huang

**Affiliations:** 1 Yellow Sea Fisheries Research Institute, Chinese Academy of Fishery Sciences; Laboratory for Marine Fisheries Science and Food Production Processes, Qingdao National Laboratory for Marine Science and Technology; Key Laboratory of Maricultural Organism Disease Control, Ministry of Agriculture and Rural Affairs; Qingdao Key Laboratory of Mariculture Epidemiology and Biosecurity, Qingdao 266071, China; dongxuan@ysfri.ac.cn (X.D.); lichen@ysfri.ac.cn (C.L.); wangyiting0223@outlook.com (Y.W.); 2 Key Laboratory of Etiology and Epidemiology of Emerging Infectious Diseases in Universities of Shandong, Shandong First Medical University & Shandong Academy of Medical Sciences, Taian 271000, China; uboat@163.com; 3 Department of Preventive Veterinary Medicine, College of Veterinary Medicine, Northwest A&F University, Yangling, Shaanxi 712100, China; 15694582011@163.com (Q.L.); sunyani@nwsuaf.edu.cn (Y.S.)

**Keywords:** Crustacea hepe-like virus 1, Hepeviridae, *Macrobrachium rosenbergii*

## Abstract

The family Hepeviridae includes several positive-stranded RNA viruses, which infect a wide range of mammalian species, chicken, and trout. However, few hepatitis E viruses (HEVs) have been characterized from invertebrates. In this study, a hepevirus, tentatively named Crustacea hepe-like virus 1 (CHEV1), from the economically important crustacean, the giant freshwater prawn *Macrobrachium rosenbergii*, was characterized. The complete genome consisted of 7750 nucleotides and had a similar structure to known hepatitis E virus genomes. Phylogenetic analyses suggested it might be a novel hepe-like virus within the family Hepeviridae. To our knowledge, this is the first hepe-like virus characterized from crustaceans.

## 1. Introduction

The family Hepeviridae belongs to positive-stranded RNA viruses and infects a wide range of mammalian species, chicken, and trout [1,2]. Due to frequent identifications of novel hepatitis E viruses (HEVs) or HEV-like viruses from various animal species, the HEV nomenclature system has been undergoing dynamic changes [3,4,5,6]. According to the consensus proposal from the International Committee on Taxonomy of Viruses (ICTV), the family Hepeviridae is currently divided into two genera: *Orthohepevirus*, including all mammalian and avian HEVs; and *Piscihepevirus*, including the Cutthroat trout virus [5,7]. Within *Orthohepevirus*, four species have been designated (A–D) that infect mammals and birds [4,8], including *Orthohepevirus* A from human, swine, deer, mongoose, rabbit, and camel; *Orthohepevirus* B from chicken and wild birds; *Orthohepevirus* C from rat, greater bandicoot, Asian musk shrew, ferret, and mink; and *Orthohepevirus* D from bats. In addition, different genotypes have also been classified based on the genomic sequence divergence within species. For example, within *Orthohepevirus* A, eight genotypes have been proposed [7]. However, to our knowledge, only a few HEVs from invertebrates have been reported, of which none were directly identified from crustaceans.

## 2. Materials and Methods

### 2.1. Sample Collection and Detection of Shrimp Pathogens

In 2018, a total of 18 diseased giant freshwater prawns (*Macrobrachium rosenbergii*) with growth retardation were collected from a farming pond in Jiangsu Province, China. Total RNA and DNA were extracted from each of these *M. rosenbergii* prawns, and the detection of known shrimp pathogens was performed using RT-PCR or PCR [9,10,11]. The shrimp pathogens included the White spot syndrome virus (WSSV), Infectious hypodermal and haematopoietic necrosis virus (IHHNV), *Enterocytozoon hepatopenaei* (EHP), Acute hepatopancreatic necrosis disease-causing *Vibrio* (AHPND-causing *Vibrio*), Taura syndrome virus (TSV), Yellow head virus genotype 1 (YHV-1), Infectious myonecrosis virus (IMNV), Decapod iridescent virus 1 (DIV1), *Macrobrachium rosenbergii* nodavirus (MrNV), and Covert mortality nodavirus (CMNV).

### 2.2. RNA Extract and Transcriptome Sequencing

Cephalothoraxes from 15 g *M. rosenbergii* were homogenized and the supernatant was centrifuged at 120,000× *g* for 4 h at 4 °C. Total RNA from viral crude extracts was extracted using TRIzol reagent (Invotrigen, Carlsbad, CA, USA) and the ribosomal RNA was removed by the Epicentre Ribo-zero^TM^ rRNA Removal Kit (Epicentre, Madison, WI, USA). Sequencing libraries were generated using the rRNA-depleted RNA by NEB Next® Ultra^TM^ Directional RNA Library Prep Kit for Illumina® (NEB, Ipswich, MA, USA), following the manufacturer’s recommendations. Then, the 150 bp paired-end sequencing of the RNA libraries were conducted using the Illumina Hiseq platform. The raw sequencing reads were adaptor- and quality-trimmed using the Trimmomatic program [12] embedded in Trinity [13]. The clean reads were directly assembled de novo using Trinity (version 2.5.1) with default parameter settings. All the assembled contigs were compared using BLASTx against the non-redundant protein database (nr) downloaded from GenBank, with an *E*-value threshold set at 1 × 10^−5^. All potential viral contigs were identified and then merged to form longer viral contigs using Geneious (version 11.1.5).

### 2.3. Viral Genome Sequencing and Sequence Analysis

To further confirm the results from next generation sequencing (NGS), RT-PCR and Sanger sequencing of viral crude extracts based on the obtained contigs were performed. Firstly, different primer pairs were designed based on the viral contigs (Table 1). Meanwhile, both the 5’ and 3’ rapid amplifications of the cDNA ends (RACE) (Invitrogen) were employed to determine the termini of the obtained viral genome. Then, the structure of the complete viral genome was analyzed by searching against the Conserved Domain Database (CDD). Both the reference sequences of the RNA-dependent RNA polymerase (RdRp) and the capsid protein were downloaded from GenBank and aligned using Mafft [14]. The conserved regions of the two alignments were obtained using Trimal [15]. Phylogenetic analysis was performed using RaxML [16], with the JTT amino acid substitution model and 1000 bootstrap replicates.

## 3. Results and Discussion

For all of the diseased giant freshwater prawns (*M. rosenbergii*), our molecular tests showed that they were negative for all known shrimp pathogens, including WSSV, IHHNV, EHP, AHPND-causing *Vibrio*, TSV, YHV-1, IMNV, DIV1, MrNV, and CMNV. However, the NGS of the viral crude extracts from the pooled samples identified one contig (size = 7696 bases) associated with Hepeviridae, with 3608 non-repetitive reads and mean depth of 60.94 ± 18.52. The first hit in the BLASTx output for this contig was AFR11848.1 (506 aa) with a sequence identity of 55%, which was a protein encoded by the opening reading frame (ORF) 2 of a *Hepelivirus* from sewage from Nepal in 2009 [17]. The second hit was ASM94024.1 (1797 aa), the polyprotein encoded by a Barns Ness breadcrumb sponge hepe-like virus 2 from *Halichondria panacea* from Scotland in 2014, with only 26% sequence identity in the helicase and RdRp regions from positions 920 to 1771. Because both *Hepelivirus* and Barns Ness breadcrumb sponge hepe-like virus 2 are currently proposed to be unclassified viruses in the Hepeviridae family, we propose that the novel viral isolate may belong to the Hepeviridae family and was tentatively named as Crustacea hepe-like virus 1 (CHEV1).

**Figure 1:**
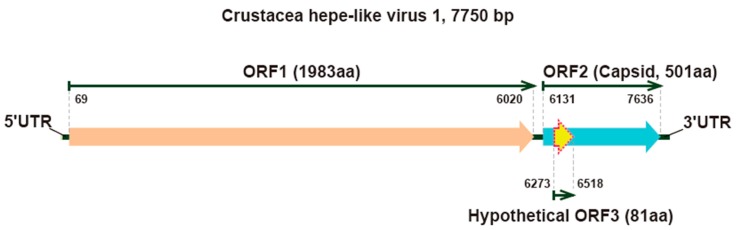
The predicted genome structure of the novel Crustacea hepe-like virus 1.

To further confirm the assembly results, RT-PCR and Sanger sequencing based on this contig were performed using primers designed based on the NGS results (Table 1). In addition, both 5’ and 3’ RACE were employed to determine the termini of the CHEV1 genome. The full genome of CHEV1 with 7750 nucleotides (nt) in length was successfully obtained and deposited in GenBank under accession number MK580123. Similar to other recognized HEVs, the newly sequenced genome also included a 5’ untranslated region (UTR), followed by two major ORFs and one hypothetical ORF, and a 3’ UTR (Figure 1). The first ORF encodes a polyprotein of 1983 amino acids (aa) from genome positions 69 to 6020 nt (Figure 1). As mentioned above, only the 3’ terminus of the polyprotein showed certain sequence identity with hypothetical helicase and RdRp of some other unclassified HEVs. The second ORF from genome positions 6131 to 7636 encoded a protein of 501 aa, which may encode the capsid protein of CHEV1 (Figure 1). The hypothetical ORF3 was predicted to be located from positions 6273 to 6518 and was fully overlapped with ORF2. It encoded a protein of 81 aa with the start codon CTG rather than ATG, which was used by the first two ORFs. It also should be noted that the hypothetical ORF3 was smaller than those of other recognized HEVs. We further compared the protein sequences of the predicted ORF3 of CHEV1 and representative HEVs [18] (Figure S1). However, these proteins were so divergent that only a few conserved amino acids could be observed. Therefore, the existence of ORF3 of CHEV1 warranted further verification. However, CHEV1 exhibited a relatively low amino acid identity to the Orthohepevirus species A Genotypes 1 (M73218, D11092, X98292, AY23020, AY204877, and JF443721) and 2 (M74506) that can infect humans [19]: The predicted ORF1, 17.3% to 17.6% for Genotype 1 and 17.5% for Genotype 2; the predicted ORF2, 14.4% to 14.7% for Genotype 1 and 13.8% for Genotype 2; and the predicted ORF3, 12.9% for Genotype 1 and 10.6% for Genotype 2.

**Figure 2:**
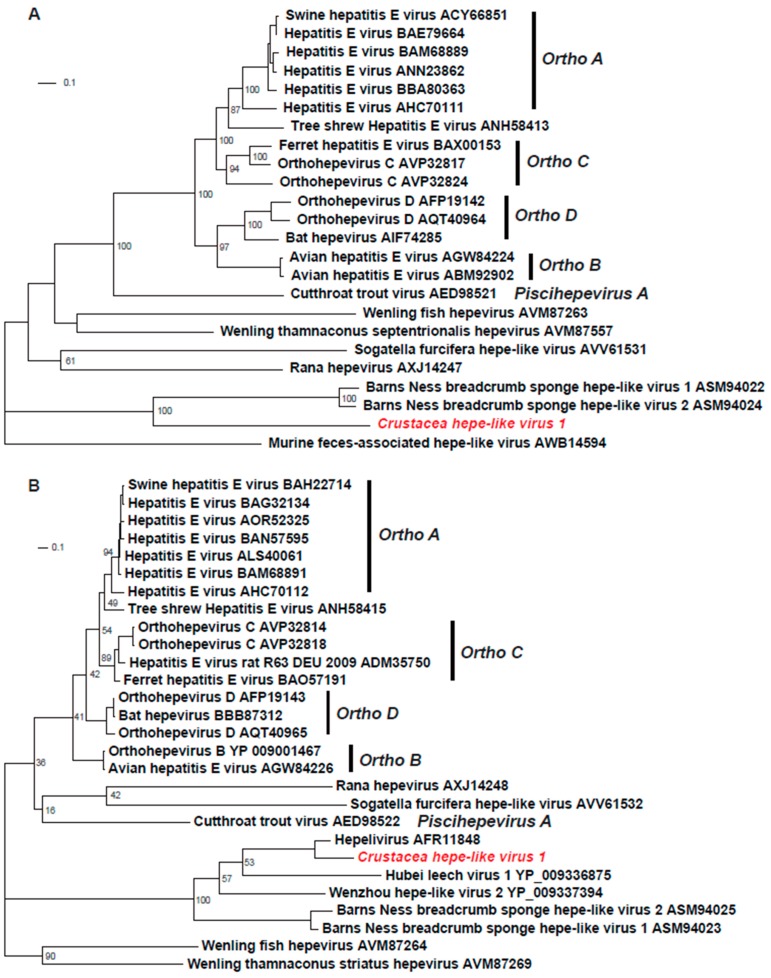
Phylogenetic analyses of the RNA-dependent RNA polymerase (RdRp) and capsid protein sequences of representative hepeviruses. (**A**) Phylogenetic analyses of RdRp sequences of representative hepeviruses. (**B**) Phylogenetic analyses of capsid protein sequences of representative hepeviruses. In both panels, *Ortho* represents *Orthohepevirus* and the novel virus is highlighted in red. The reference sequences were downloaded from GenBank and aligned using Mafft. Phylogenetic analysis was performed using RaxML, with the JTT amino acid substitution model and 1000 bootstrap replicates.

In order to study the phylogenetic position of CHEV1, the RdRp and capsid protein sequences were extracted and phylogenetically analyzed with those of representative HEVs. In both trees, the HEVs clustered into two major branches, which basically represented HEVs infecting vertebrates and invertebrates, respectively. The branch of vertebrate hepeviruses included the two previously classified *Orthohepevirus* and *Piscihepevirus* genera, as well as several unclassified species from fish, whereas CHEV1 was located in the branch of invertebrate hepeviruses (Figure 2). Consistent with the previous BLASTx search, phylogenetic analysis of the RdRp protein sequences showed that CHEV1 clustered with Barns Ness breadcrumb sponge hepe-like viruses 1 and 2, despite with a very long branch separating them (Figure 2A). In the tree constructed using the capsid proteins, CHEV1 formed a separate cluster with *Hepelivirus* (Figure 2B). Remarkably, CHEV1 did not fall within the two classified genera, and hence may represent a novel hepe-like virus in the family Hepeviridae (Figure 2).

## 4. Conclusions

In summary, we described a novel hepe-like virus from diseased *M. rosenbergii*, which possessed two major ORFs and a hypothetical ORF3 and might represent a novel hepe-like virus in the family Hepeviridae. To our knowledge, it is the first hepe-like virus identified from crustaceans. Although we could not determine the novel virus as the causative agent for the outbreak based on current evidence, our study highlighted the expanding host range and the increasing species diversity of the family Hepeviridae.

## Figures and Tables

**Table 1:** Primers used for amplification and sequencing of the Crustacea hepe-like virus 1 genome.

Primer	Sequence 5’-3’
HE-1F	TGATAACGATGGATATTAATCCACAT
HE-1R	AGAAGTTGAAAATGCCGCTGAT
HE-2F	TCGTTTTCAAGAAAGGCAACAA
HE-2R	TAGAGGGAGCATGACTGGTTTGT
HE-3F	TTCACGTATGTCCAACACAATAACTA
HE-3R	GTGGGGTTGGTCTTATAGCGTA
HE-4F	ACTTCTTGGACATCCATTCCACA
HE-4R	GTGTAAAGATGTCTTACTTGCTCTGTT
HE-5F	GAAAACATCCACGCTCAAAATC
HE-5R	GGTTCATACCATTCTTTCTCCAGTT
HE-6F	CAAGCTAAGAAGCAATGCCGT
HE-6R	TTGTTCCAAGTCCTCAGGTGTGT
HE-7F	ATACATCTCCAACATTGTCACACCA
HE-7R	CTTTGAGTGCGTTGGCAATGT
HE-8F	GGAAAGCCTGGGACAGAGATA
HE-8R	ATTGCGATACTATGTAGGCCCA
5’adaptor	GCTGTCAACGATACGCTACGTAACGGCATGACAGTGCCCCCCCCCCCCCCC
3’adaptor	GCTGTCAACGATACGCTACGTAACGGCATGACAGTGTTTTTTTTTTTTTTTTTT
5.3’outer	GCTGTCAACGATACGCTACGTAAC
5.3’inner	GCTACGTAACGGCATGACAGTG
CHEV-H-F1	CGTCCAGGAAAGGGCCAGTTAACAC
CHEV-H-F2	CGATCAAAATCGATGGGGCCTAAAGT
CHEV-H-R2	GCTGCAGATATTTGATCCCTGTGTCGTT
CHEV-H-R1	ATTGGTCGACGATAAGTGGATCATTGTTCATA
CHEV-H-RT2	TTGACTGCGTAAGCGTAA
CHEV-H-RT1	GAGGTATTAGGTTGATGTCG

## Supplementary Materials

Supplementary materials can be found at https://www.mdpi.com/1999-4915/12/3/323/s1.

## References

[1] Sooryanarain H., Meng X.J. (2019). Hepatitis E virus: Reasons for emergence in humans. Curr. Opin. Virol..

[2] Lara J., Purdy M.A., Khudyakov Y.E. (2014). Genetic host specificity of hepatitis E virus. Infect. Genet. Evol..

[3] Smith D.B., Simmonds P. (2018). Classification and Genomic Diversity of Enterically Transmitted Hepatitis Viruses. Cold Spring Harb. Perspect. Med..

[4] Smith D.B., Purdy M.A., Simmonds P. (2013). Genetic variability and the classification of hepatitis E virus. J. Virol..

[5] Smith D.B., Simmonds P., Jameel S., Emerson S.U., Harrison T.J., Meng X.J., Okamoto H., Van der Poel W.H., Purdy M.A. (2014). Consensus Proposals for Classification of the Family Hepeviridae. J. Gen. Virol..

[6] Oliveira-Filho E.F., Konig M., Thiel H.J. (2013). Genetic variability of HEV isolates: Inconsistencies of current classification. Vet. Microbiol..

[7] Pavio N., Meng X.J., Doceul V. (2015). Zoonotic origin of hepatitis E. Curr. Opin. Virol..

[8] Okamoto H. (2007). Genetic variability and evolution of hepatitis E virus. Virus Res..

[9] Chen J., Wang W., Wang X., Zhang Q., Ren Y., Song J., Wang X., Dong X., Huang J. (2018). First detection of yellow head virus genotype 3 (YHV-3) in cultured Penaeus monodon, mainland China. J. Fish Dis..

[10] OIE (2018). Manual of Diagnostic Tests for Aquatic Animals.

[11] Qiu L., Chen M.M., Wan X.Y., Li C., Zhang Q.L., Wang R.Y., Cheng D.Y., Dong X., Yang B., Wang X.H. (2017). Characterization of a new member of Iridoviridae, Shrimp hemocyte iridescent virus (SHIV), found in white leg shrimp (Litopenaeus vannamei). Sci. Rep..

[12] Bolger A.M., Lohse M., Usadel B. (2014). Trimmomatic: A flexible trimmer for Illumina sequence data. Bioinformatics.

[13] Grabherr M.G., Haas B.J., Yassour M., Levin J.Z., Thompson D.A., Amit I., Adiconis X., Fan L., Raychowdhury R., Zeng Q. (2011). Full-length transcriptome assembly from RNA-Seq data without a reference genome. Nat. Biotechnol..

[14] Nakamura T., Yamada K.D., Tomii K., Katoh K. (2018). Parallelization of MAFFT for large-scale multiple sequence alignments. Bioinformatics.

[15] Capella-Gutierrez S., Silla-Martinez J.M., Gabaldon T. (2009). trimAl: A tool for automated alignment trimming in large-scale phylogenetic analyses. Bioinformatics.

[16] Stamatakis A. (2014). RAxML version 8: A tool for phylogenetic analysis and post-analysis of large phylogenies. Bioinformatics.

[17] Ng T.F., Marine R., Wang C., Simmonds P., Kapusinszky B., Bodhidatta L., Oderinde B.S., Wommack K.E., Delwart E. (2012). High variety of known and new RNA and DNA viruses of diverse origins in untreated sewage. J. Virol..

[18] Smith D.B., Simmonds P., Izopet J., Oliveira-Filho E.F., Ulrich R.G., Johne R., Koenig M., Jameel S., Harrison T.J., Meng X.J. (2016). Proposed reference sequences for hepatitis E virus subtypes. J. Gen. Virol..

[19] Doceul V., Bagdassarian E., Demange A., Pavio N. (2016). Zoonotic Hepatitis E Virus: Classification, Animal Reservoirs and Transmission Routes. Viruses.

