# Supplementary figures and images for: A Novel Hepe-Like Virus from Farmed Giant Freshwater Prawn Macrobrachium rosenbergii
Source: Viruses. 2020 Mar 17;12(3):323. doi: 10.3390/v12030323 (PMC7150978; doi:10.3390/v12030323)

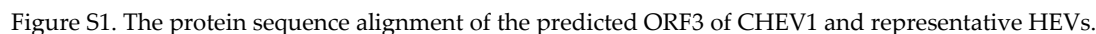

Supplement: Supplementary file 1 [file viruses-12-00323-s001.pdf]
